# A Case of Hypertriglyceridemia-Induced Acute Pancreatitis in the Setting of Alcohol Abuse

**DOI:** 10.7759/cureus.38028

**Published:** 2023-04-23

**Authors:** Samuel Nwaobi, Ayesha Khan, Pius E Ojemolon, Amaka C Ugoh, Blessing C Iheme

**Affiliations:** 1 Family Medicine, Piedmont Columbus Regional-Midtown, Columbus, USA; 2 Internal Medicine, Edward Via College of Osteopathic Medicine, Auburn, USA; 3 Internal Medicine, John H. Stroger, Jr. Hospital of Cook County, Chicago, USA; 4 Internal Medicine, University of Benin Teaching Hospital, Benin, NGA; 5 Family Medicine, American University of Barbaods, School of Medicine, Bridgetown, BRB

**Keywords:** obesity, alcohol addiction, pancreatitis, severe hypertriglyceridemia, alcohol use disorder (aud)

## Abstract

Acute pancreatitis (AP) is the painful inflammation of the pancreas. It is commonly associated with gallstones, excessive alcohol use, and certain medications. We report a case of hypertriglyceridemia-induced pancreatitis in a 35-year-old African American male with a history of alcohol abuse, tobacco use, and hyperlipidemia who presented with abdominal pain and intractable vomiting. During history taking, he reported chronic alcohol abuse over the past 10 years. On physical examination, he was ill-looking, with a dry mucous membrane and reproducible epigastric tenderness. Laboratory testing indicated markedly elevated triglycerides and lipase levels. Computed Tomography imaging showed signs of pancreatic inflammation. He was treated with aggressive intravenous fluid hydration, insulin infusion, and pain control medications. He demonstrated significant improvement and then transitioned to oral fibrates. Community resources for alcohol abuse treatment were provided and a referral was made to endocrinology for outpatient follow-up. This case highlights acute pancreatitis in a person with high alcohol use with elevated triglyceride and explores possible associations between these three.

## Introduction

Acute pancreatitis (AP) is a potentially fatal condition and has been reported as one of the most frequent gastrointestinal causes of hospital admissions in the United States [1]. The most common causes of AP are alcohol misuse and gallstone pancreatitis [2]. Although hypertriglyceridemia (HTG) is not considered a common cause, it is a well-established etiology of AP, as it accounts for 2-4% of cases of AP [3]. The incidence of acute pancreatitis has increased from 27.6 per 100 000 population in 1999 to 35.9 per 100 000 in 2010 with a mean annual increase of 2.7% per year over the 12-year period [1,4]. About one-third of acute pancreatitis cases in the United States are alcohol-induced, and 60%-90% of pancreatitis patients have a history of chronic alcohol consumption [5]. Chronic alcohol consumption is thought to be responsible for 17% to 25% of acute pancreatitis cases worldwide. Pancreatitis rarely occurs following a single binge-drinking episode but is usually found in patients with a history of four to five drinks daily over five years of ongoing use [6]. Excessive alcohol use disorder is associated with high morbidity and mortality. Heavy drinkers are at risk for cardiovascular disease, liver disease, pancreatitis, gastritis, esophagitis, chronic infections, and many more illnesses [7]. Hypertriglyceridemia can be attributed to either primary or secondary causes. Primary causes include familial dyslipidemias such as familial chylomicronemia, familial HTG, and mixed HTG. These often result in severe hypertriglyceridemia (>1000 mg/dL). Secondary causes include being overweight, excessive alcohol consumption, uncontrolled diabetes mellitus, hypothyroidism, chronic kidney disease, and medications such as estrogens, corticosteroids, and retinoids. Serum triglyceride (TG) levels >1000mg/dL are typically required for HTG to be considered the underlying etiology of acute pancreatitis [2,8]. We present a case of acute pancreatitis likely caused by hypertriglyceridemia in a patient with a history of alcohol use disorder. This report explores the relationship between chronic alcohol use in triglyceride elevation and the subsequent development of HTG-induced acute pancreatitis. 

## Case presentation

A 35-year-old African American man with a history of heavy alcohol use, and tobacco abuse presented for evaluation of acutely worsening epigastric pain for two days and multiple episodes of vomiting with decreased oral intake. The patient was unaware of any relieving or exacerbating factors. The epigastric pain did not radiate. He reported a history of alcohol use averaging 41 standard drinks weekly. He admitted to binging on alcohol during the holidays in the week before the presentation. He does not have any known family history of hyperlipidemia. He denied any prior surgeries or medication use. He also denied fever, headache, chest pain, or shortness of breath. 

**Figure 1:**
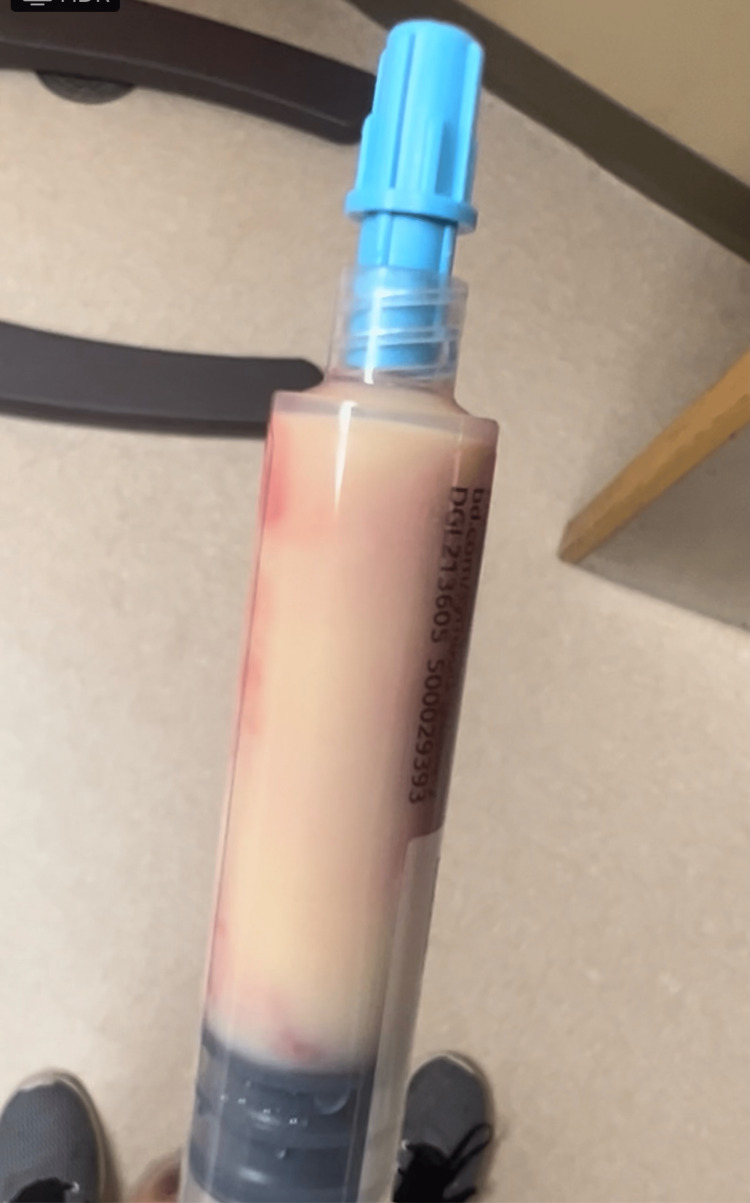
Lipemic blood sample

On presentation, he was frail (weight = 59kg, BMI = 19.2kg/m2) and in acute distress due to severe abdominal pain. He was afebrile and tachycardic with a pulse of 117 beats/minute and normal oxygen saturation on room air. Physical examination revealed dry mucous membranes, normal cardiac and pulmonary exam findings, positive epigastric tenderness, and voluntary guarding of his epigastric region. His alcohol use disorders identification test (AUDIT-C) screen at the time of admission indicated hazardous alcohol use with a score of 8 (a score of 0 suggests no alcohol use, a score of <3 suggests a normal alcohol consumption pattern, and scores greater than four in men and greater than three in women indicate alcohol misuse). The patient had blood drawn for laboratory investigations including a complete blood count (CBC), comprehensive metabolic panel (CMP), serum lipase levels, and a lipid panel. On visual inspection of the specimen, it was noted to be thick and whitish (Figure 1). White blood cell (WBC) count was 12, 200 cells/µL (reference range: 4,400 - 10, 600 U/L). The CMP showed elevated alanine transaminase (ALT), aspartate transaminase (AST), and total bilirubin levels (Table 1). Lipid panel results showed high triglyceride levels > 4000 mg/dl (Table 2). Serum lipase was 1,039 U/L (Reference range: 73 - 393 U/L). The patient's calculated bedside index for severity in acute pancreatitis (BISAP) score was one point indicating a lower risk of in-hospital mortality.

**Table 1:** Comprehensive Metabolic Panel showing elevated Liver Enzymes

Investigation	Result	Reference range
Sodium	138 mmol/L	136 - 145 mmol/L
Potassium	4.4 mmol/L	3.5 - 5.2 mmol/L
Chloride	102 mmol/L	98 - 108mmol/l
CO2	26 mmol/L	21 - 31mmol/l
BUN	15 mg/dL	5 - 27 mg/dL
Creatinine	1.32 mg/dL	0.70 - 1.30 mg/dL
Calcium	8.4 - 10.2mg/dL	7.6 mg/dL
Glucose	134 mg/dL	65 - 99 mg/dL
Total protein	6.8g/dL	6.0 - 8.5g/dL
Albumin	2.8 mg/dL	3.5 - 5.0 g/dL
Albumin/Globulin Ratio	0.7	1.0 - 2.0
Total bilirubin	1.8 mg/dL	<1.2 mg/dL
Calcium	7.6 mg/dL	8.4 - 10.2 mg/dL
AST	155 IU/L	<42 U/L
ALT	89 IU/L	6 - 52 U/L
Alkaline Phosphatase	98	35 - 125 U/L
Anion Gap	14	10 - 17
BUN/Creatine Ratio	11	8 - 20

**Table 2:** Lipid panel showing hyperlipidemia

Component	Reference Range and Units	Value
Total cholesterol	≤ 200 mg/dl	510mg/dl
Triglycerides	2 - 150 mg/dl	> 4,000mg//dl
High-density Lipoproteins (HDL)	≥ 40.00mg/dl	25.00mg/dl
Cholesterol/High-density lipoprotein ratio (Chol/HDL)	0.00 - 5.00	20.40

**Figure 2:**
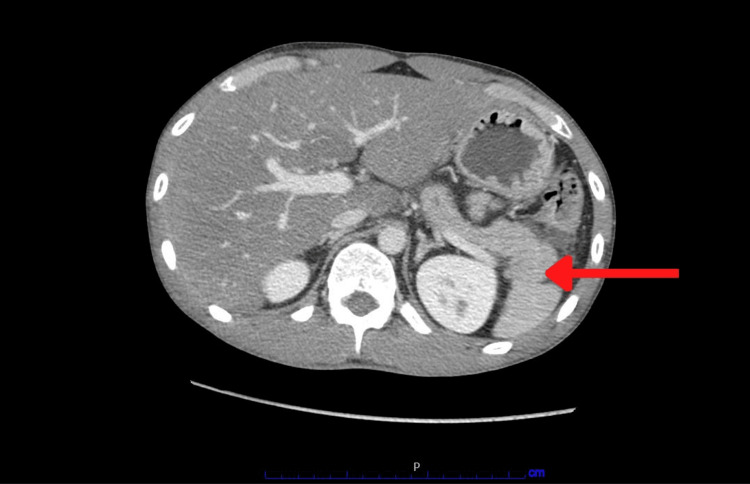
Computed tomogram of the abdomen and pelvis showing acutely inflamed pancreatic parenchyma.

He underwent computed tomography of the abdomen and pelvis with intravenous (IV) contrast which showed evidence of hepatic steatosis, enlarged edematous appearing pancreatic head with extensive fluid surrounding the right abdomen consistent with acute pancreatitis, no gallstones seen, and normal biliary ducts (Figure 2). 

Based on the patient's tachycardia and white blood cells (WBC) he met the criteria for systemic inflammatory response syndrome (SIRS). However, with clear evidence of acute pancreatitis, sepsis was a less likely diagnosis. He was treated with IV fluids given via a 3L normal saline bolus followed by 150 mL/hour for maintenance. He was commenced on IV pain management and enteral feeding as tolerated. Given the severe elevation in his TG, he was started on an insulin infusion with a target glucose of <180 mg/dL and TG of <500 mg/dL. Once the TG levels were below 500 mg/dl, he was transitioned from IV insulin to oral gemfibrozil. His triglycerides decreased to 229 U/L and his AST and ALT normalized prior to discharge. A subsequent outpatient referral to a primary care physician and endocrinologist was made for appropriate follow-up. Below is a table that shows a downward trend in his triglyceride levels (Table 3).

**Table 3:** Triglyceride downward trend over the course of treatment

Triglyceride downward trend
>4,000
1,096
335

## Discussion

Alcohol abuse is prevalent in the US and worldwide [7]. Its harmful effect on health is widely recognized. A study into the effect of alcohol consumption, on multiple lipid types in the large bi-ethnic population of atherosclerosis risk in communities study showed greater levels of alcohol consumption result in significantly greater levels of triglycerides [9]. Plasma triglycerides can be exogenous in origin (i.e., from dietary fat) and carried in chylomicrons, or endogenous (from the liver) and carried in very-low-density lipoprotein (VLDL) particles [10].

A study conducted with elderly Korean men showed an increase in HTG prevalence from 28.8% to 35.7%, highlighted in individuals consuming 5.0 to 14.9 grams of alcohol each day. In this same study, they also recognized a higher prevalence increase of 38.9% in men who consumed more than 30 grams of alcohol in a day [11, 12]. Another study conducted among a group of Spanish individuals showed a three-fold increase in plasma triglycerides in those who had alcohol twice during the weekend compared to non-drinkers, with a mean and standard deviation report of 0.52 ± 0.14 mmol/l in drinkers versus 1.51 ± 0.25 mmol/l in non-drinkers [13].

The consumption of alcohol stimulates fat ingestion, thus increasing chylomicrons secretion from the small intestine. After these chylomicrons are secreted, they are taken into the liver leading to a high free fatty acid influx until there is an upregulation of microsomal triglyceride transfer protein (MTP) followed by secretion of VLDL. The increased number of VLDL and intermediate-density lipoproteins (IDL) causes increased competition for LDL leading to a higher increase in triglyceride. Acute heavy alcohol intake combined with a fatty meal may induce hypertriglyceridemia due to an increase in VLDL secretion, impaired lipolysis, and increased hepatic delivery of free fatty acids (FFA) from adipose tissue through FFA transporters such as CD36. Note that, while an acute consumption of alcohol causes impairment of lipolysis via lipoprotein lipase, chronic consumption of alcohol causes the opposite effect, which is the up-regulation of lipoprotein lipase (LPL) [11-13].

The SHIBA (severe hypertriglyceridemia influenced by alcohol) syndrome is an interesting article published in Alcohol and Alcoholism which states that chronic alcohol consumption patients with a combination of obesity, diabetes, and alcohol abuse are prone to develop extremely high TG values [14]. Elevated triglycerides are associated with the development of pancreatitis, but the exact mechanism remains unknown. There are two proposed theories on the mechanism of HTG-induced pancreatitis. One focuses on the role of chylomicrons, and the other on the impact of excess FFA in circulation.

Chylomicrons are TG-rich lipoprotein particles produced in enterocytes from dietary lipids composed of a main central lipid core that consists primarily of TGs and carry esterified cholesterol and phospholipids [15]. They are usually seen in circulation when serum TG levels exceed >10mmol/L [16]. The chylomicrons are believed to be responsible for pancreatic inflammation by impairing circulatory flow in capillary beds resulting in ischemia. Chylomicrons increase the viscosity of plasma leading to capillary plugging, ischemia, and acidosis in acinar cells which eventually triggers acute pancreatitis [16-20]. Pancreatic lipase is responsible for the hydrolysis of chylomicrons. High levels of FFA are released via the hydrolysis of chylomicrons in the vascular bed of the pancreas.

Note that these high levels of FFA released, exceed the binding capacity of plasma albumin. This unbound FFA self-aggregates into micellar structures with detergent properties and pro-inflammatory properties which causes damage to platelets, vascular endothelium, and pancreatic acinar cells (Figure 4).

**Figure 3:**
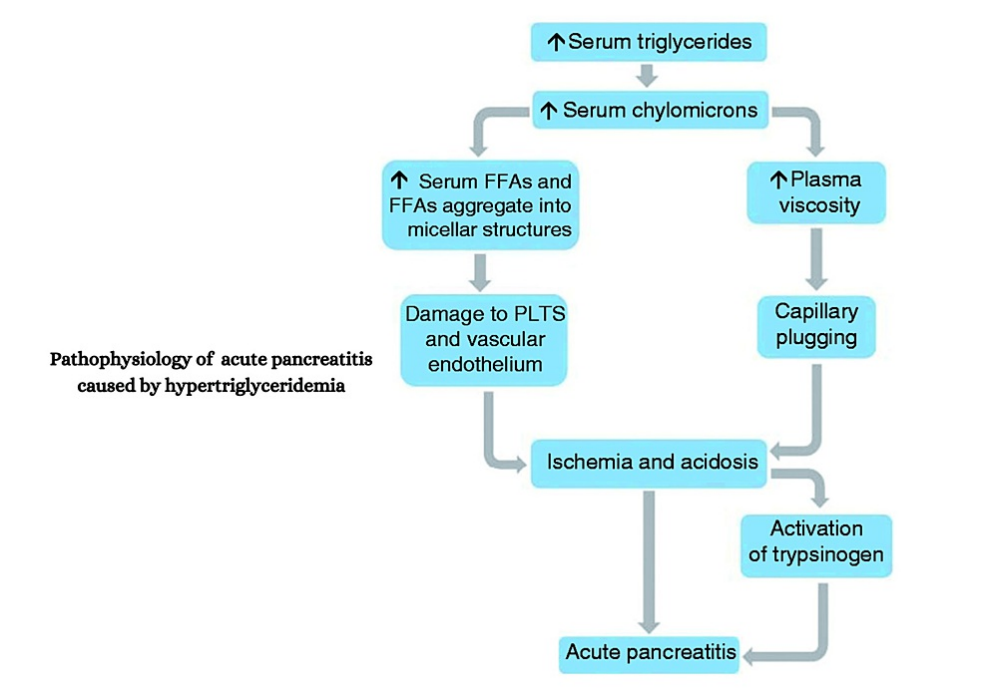
Pathophysiology of Acute Pancreatitis caused by Hypertriglyceridemia

Ischemia and acidosis follow after this damage eventually activating trypsinogen which triggers acute pancreatitis via necrosis, edema, and inflammation [1,16-20].

Patients with HTG-induced pancreatitis are often treated acutely with insulin, fluids, heparin, and in some cases plasmapheresis if it is available at the medical facility [3]. If insulin infusion treatment is given, frequent blood glucose checks should be done to avoid hypoglycemia, and if needed, dextrose infusion should be started to maintain euglycemia. Once the acute pancreatitis attack is abated and triglyceride levels have decreased, lipid-lowering medication such as fibrates should be added to achieve long-term TG control below 500 mg/dl. Patients should also be counseled by a dietician on a low-fat, low-sugar diet [3].

In the long term, patients should avoid medications such as Clozapine, Olanzapine, and Corticosteroids are known to increase TG levels as well as possible triggers. Alcohol cessation must be encouraged, and brief alcohol intervention is recommended when admitted to the hospital or during their primary care follow-up visit [6].

## Conclusions

Elevated triglyceride levels and Alcohol Use Disorder both pose significant health risks. It is essential to monitor lipid levels in patients with excessive alcohol intake and initiate early treatment if necessary to avoid severe complications of hypertriglyceridemia such as acute pancreatitis. 
